# What has the COVID-19 pandemic taught us about conducting patient and public involvement remotely? Insights from a series of digital meeting observations

**DOI:** 10.1186/s40900-021-00315-9

**Published:** 2021-10-11

**Authors:** Elin Lampa, Björn Sonnentheil, Antónia Tökés, Georgina Warner

**Affiliations:** 1 Child Health and Parenting (CHAP), Department of Public Health and Caring Sciences, Uppsala University, BMC, Husargatan 3, Box 564, 751 22 Uppsala, Sweden; 2 Public Contributor, Borlänge, Sweden

**Keywords:** Patient and public involvement, COVID-19, Remote work, Digital, Observation, Group dynamics

## Abstract

**Background:**

During the COVID-19 pandemic many work tasks are being done remotely through digital meetings, including PPI in research. Yet, some PPI activities have been paused or cancelled altogether during the pandemic. In this commentary, we share our insights from observing digital meetings with researchers and public contributors, representing vulnerable groups. Additionally, we discuss how remote PPI activities can be understood and improved.

**Main body:**

As part of a PPI evaluation project, live observations were conducted by two trained observers, using a semi‐structured observation protocol developed to objectively assess aspects of group dynamics in PPI research meetings with public contributors with experience of seeking refuge and parents facing economic hardship. This project’s data collection is ongoing and the insights in this commentary is based on the observers’ discussion. We discuss these insights through the lens of the Media Richness Theory, stating that the choice of media we communicate through should be guided by what kind of information we want to communicate to each other. The more complex the information is, the richer the media tool needs to be. For example, information in a text message is more easily misinterpreted than information given in person. This is because meeting in person gives us more information, for example through body language and tone of voice. Based on our experiences from observing digital research meetings, we give suggestions on how to improve digital meetings with public contributors. A few key points are: actively choosing which media to use; being prepared to guide contributors to the chosen media in a way that is suitable for them; and the increased importance of the person chairing the meeting to actively include all participants.

**Conclusions:**

We reach the conclusion that digital meetings with public contributors is possible, but that researchers need to make a commitment and actively work to solve practical issues. Finally, the format and structure of digital meetings should be co-created together with public contributors.

## Plain English summary

During the COVID-19 pandemic many work tasks are conducted remotely through digital meetings, including PPI in research. Yet, some PPI activities have been paused or cancelled during the pandemic. In this commentary, we share our insights from observing digital meetings with researchers and contributors with experience of seeking refuge and parents facing economic hardship, and discuss how remote collaborations can be improved. This is discussed through the lens of the Media Richness Theory, stating that the choice of media we communicate through should be guided by what kind of information we want to communicate to each other. The more complex the information is, the richer the media tool needs to be. For example, information in a text message is more easily misinterpreted than information given in person, since meeting in person gives us more information, for example through body language and tone of voice. Based on our experiences from observing digital research meetings, we give suggestions on how to improve digital meetings with public contributors. Some key points are: actively choosing which media to use; being prepared to guide contributors to the chosen media in a way that is suitable for them; and the increased importance of the person chairing the meeting to actively include all participants. We reach the conclusion that digital meetings with public contributors is possible, but that researchers need to make a commitment and actively work to solve practical issues. Finally, the format and structure of digital meetings should be co-created together with public contributors.

## Background

This commentary sets out to discuss insights on remote Patient and Public Involvement (PPI), conducted through digital meetings, gained from observations in a PPI evaluation project. PPI in research, here defined as the active research partnership between patients and/or representatives of the public and researchers [8], has in recent years been increasingly recognised for its potential to contribute significantly to all stages of the research process [9–11]. In the PPI literature, recognising context and avoiding tokenistic approaches is highlighted as essential for and meaningful and effective PPI [12–14]. However, most reports on successful PPI activities were published before the pandemic.

During the ongoing pandemic, many workplaces have moved from in-person meetings to remote work through digital meetings in line with restrictions to minimize spread of the corona virus. Before the pandemic most work places, including research teams, did not have the practical experience of remote work to the extent they do now. Our experiences indicate that many research teams have discovered that research activities often can be done remotely. The fast development of digital tools, even before the pandemic, has most likely facilitated the transition to remote work.

As with other work meetings, we can expect changes in PPI activities as consequences of the pandemic. Some activities might have been postponed or cancelled entirely, while other activities have been conducted remotely, i.e. not through in-person meetings but through video conferences or other digital communication tools. When attempting to find literature for this commentary only one example of digital PPI activities could be identified. In this report, researchers evaluated a digital collaboration forum which aimed to facilitate remote PPI. The forum was experienced as functional, feasible and acceptable, but users suggested it should be less researcher-focused and better meet the needs of public contributors [15]. There are, however, most likely several projects that have conducted remote PPI recently, but not yet reported on it.

Even though there are clear benefits to remote work, like aspects on health and climate [1], we need to be aware of how this affects our teams and collaborations over time. Switching from in-person to remote working in research is likely to have unexpected—positive as well as negative—effects on the outcomes from meetings and collaborations [2], and hence on the research we produce. According to the media richness theory (MRT), developed in the 1980s by organizational scholars Daft and Lengels [3], the medium we use for communication has an effect on the richness of the information we transmit to each other and thereby on the understanding of the person at the receiving end [3]. Using MRT, communication media can be ranked and evaluated according to its ability to communicate rich information. Typically, text messages would not be considered communicating rich information, while meeting in person is a rich communication media that allows for the transmission of social cues, eye contact and body language. This is especially relevant when communicating complex messages. To decide on a time for a meeting, a text message or email might be sufficient. But, to take decisions in a research process, where individuals with different backgrounds and skill sets are involved, rich information transmission is important for mutual understanding and correct interpretations, in both directions. The fast-paced technological development since the 1980s has of course put this theory in another light, but the core ideas remain intact [4].

However, collaboration is not restricted to transmitting information, but factors such as social interaction and group dynamics are highly affecting collaboration [5]. One specific aspect which is important for solving tasks that require interpersonal skills is the capability of the communication media to convey *social presence,* i.e. if participants perceive others as psychologically present. In general, richer media formats enable higher social presence and are considered more personal [3]. One study showed that in virtual teams, the use of a richer communication media improved team cohesion by decreasing perceived social loafing [6]. Another study adds that rich media communication is even more important for a team early in their collaboration [7].

The insights in this commentary stem from our experiences in an ongoing PPI evaluation project. In this project, we observe PPI research meetings in other research projects. Through the recruitment efforts in this study, it has become clear that researchers are postponing or even canceling PPI activities during the pandemic—especially when involving representatives from vulnerable populations, such as the refugee population. We would like to argue that this is unnecessary. Although exploring remote PPI conducted through digital meetings was not the focus of the mentioned PPI evaluation project, conducting these observations has led us to the conclusion that digital remote collaborations can be achieved with contributors from vulnerable groups—with adjustments. We think it is essential to try to maintain PPI activities throughout the projects, as important decisions are taken even during the pandemic. However, when conducting remote PPI through digital meetings, we need to be aware that digital communication, such as during video conferences, poses a different set of challenges than in-person communication. Additional efforts will be required from the researchers, to reach the same level of input, information sharing and collaboration.

## Context

This commentary is based on observations of PPI research meetings, conducted as part of the ongoing PPI evaluation project*.* In this project, we conducted behavioural observations of PPI research meetings conducted within other research projects, involving vulnerable groups. The observed research meetings were therefore held as part of three separate research projects; two refugee child mental health trials [16, 17], which involved parents with experience of seeking refuge, and one pilot project testing an intervention with financial counselling for parents, which involved parents facing economic hardship. The projects involved the contributors, together with professional stakeholders and external research advisors, in all research meetings where major decisions on the projects were taken. In addition, one of the refugee child mental health trials also involved refugee youth. The common factor for these projects was that the public contributors all represented vulnerable groups. In total, five in-person meetings and four digital meetings, on Zoom, were observed. Zoom is a cloud-based video communications app. The functionality includes chat, annotation, whiteboarding, and breakout rooms. It has dynamic voice detection whereby the screen indicates the active speaker, either through primary camera view or the edge of the participant’s camera view highlighted in yellow depending on the individual’s view selection. In the observed meetings, breakout rooms was the only one of the mentioned functionalities which was used, on one occasion in one meeting.

These PPI research meetings were observed, using a semi‐structured observation protocol, developed to objectively assess aspects of group dynamics in PPI research meetings [18]. The observation protocol consists of positive and negative observable behaviours: interpersonal relations between researchers and contributors; nature of their contributions; how contributors guided research development. Additionally, the observation protocol allows for the observers to take notes on examples of the observed behaviours and includes a standardised format for recording the meeting context. The observations were conducted live by two trained observers, who discussed their notes with each other after each meeting. This data collection is still ongoing. Utilising the observation protocol in our ongoing research has, however, enabled us to consider aspects that could be important when conducting remote PPI, leading to the insights presented in this paper.

Initially, all observations within the PPI evaluation project were conducted in in-person meetings, but the pandemic forced meetings to rely on digital solutions. We then considered whether it was possible and meaningful to use the observation protocol in digital meetings with PPI representatives. Even though the group dynamics can be expected to change in a digital room, compared to in-person, we were uncertain whether there would be enough observable interactions between meeting participants to be able to assess the PPI activity. We decided to test the feasibility of the observation protocol in digital research meetings with PPI contributors, followed by a discussion between the observers on each item, whether it could be observed or how observations differed. From this, we reached the conclusion that the observation protocol can be used in digital meetings. In addition, the observers discussed and summarised the observed differences between in-person PPI meetings and digital PPI meetings, contributing to this paper.

## Our insights from remote PPI conducted through digital meetings

### Less spontaneous interactions

We observed a reduction in the number of spontaneous positive interactions in the digital meetings. This could be observed as an increased silence before the meetings had officially started, a time in in-person meetings that is often used for spontaneous interactions between individuals. We observed an increase in the amount of direction from the meeting chair, indicating that they had to work harder to facilitate positive interactions. Additionally, interactions were often interrupted by new meeting participants joining the digital meeting. Previously, less structured elements of the research meeting, such as coffee breaks, offered observable insights to the PPI dynamics, whereas the digital meetings that have been observed so far have not included this aspect. Instead, breaks were taken individually away from the screen. This removes the possibility to observe these moments and is likely to offer less opportunities for spontaneous interactions. In addition, we saw a difference between groups that had met in-person extensively prior to working remotely and those that were formed shortly before the transition. This suggests that it might be an increased problem if the group does not have an established in-person relationship before starting to work remotely.

### The lack of non-verbal cues

When observing digital meetings, non-verbal cues were more difficult to observe and interpret. This was both related to the limited view of other meeting participants in digital video meetings, which is limited to the face or upper body, with variations in video quality, and that meeting participants cannot turn towards each other as they would have in a room. In an in-person meeting, subtle gestures like making eye contact or turning in a person’s direction can signal an interest in their opinion and include them in the discussion. In a digital meeting, these discrete cues are more difficult to pick up on and we rely more on verbal communication. We observed less non-verbal invitations to including another meeting participant in the discussion, which we interpret as related to the digital format. Instead, more direct questions were asked and the meeting chair was more active in guiding the discussions, which was related to more structure and less dynamic discussions.

### Increased linguistic barriers

Different forms of linguistic barriers to participation might increase in digital meetings. In the observed meetings, some contributors did not speak Swedish, the majority language of the context, thus interpreters were hired. Selecting suitable interpreters had required substantial considerations when organizing in-person meetings. When comparing observations of in-person meeting and digital meetings, the challenges with language interpretation seemed to be exacerbated in a digital meeting. Larger meetings, ten participants or more, in particular increased the obstacles for good language interpretation. This led one project team to opt for smaller meetings, in which one or two members of the research team met with PPI contributors. In another observed meeting, contributors were considered proficient enough in the majority language when meeting in person, but in the digital format we observed signs that communication was more challenging. For example, the contributors participated less in discussions and asked for questions to be repeated more often. In relation to this, in digital meetings it might be more difficult for the meeting chair to pick up if contributors miss out on information due to linguistic barriers or researchers’ jargon, and thus more difficult to solve potential linguistic issues.

### Difficulties to claim space in a digital room

One important aspect for public contributors’ ability to contribute to a digital meeting is how comfortable they feel sharing their input in a meeting. We have observed that public contributors share less information in digital meetings, and this can be related to that it might be more difficult to claim your space in a digital room. When using video conferencing platforms, taking turns to speak is more pronounced, which means less spontaneity. Additionally, participants always need to speak to the entire group, which can be discouraging and might cause people to hesitate before speaking up. One project team opted for smaller meetings with the contributors and just a few researchers, which seemed to alleviate this issue.

### The changing role of the meeting chair

The role of the meeting chair is always important to guide the meeting, facilitate discussion and make sure meeting participants are included. However, in digital meetings we have observed that the meeting chair tends to take on a more active and directive role, and appears to have an increased responsibility for the active inclusion of all meeting participants. Not only has the meeting chair had more speaking time in the observed digital meetings, they have more actively guided the discussion and invited meeting participants into the discussion more often. More specifically, the meeting chair, and sometimes other researchers in the meeting, has invited the public contributors to share their views more often.

In many video conferencing systems, it can be difficult to see all meeting participants at the same time, especially in larger meetings. When a person is quiet in a digital meeting, the other meeting participants do not necessarily notice their absence the same way they would have in a room. This leaves the meeting chair with larger responsibility to actively include all meetings participants in discussions.

### We need to see each other’s faces

Opting for a video conference instead of a phone meeting provides more information sharing between meeting participants, as we can see each other’s faces. In the observed digital meetings, most meeting participants had their cameras turned on. When meeting participants instead had their camera turned off during a meeting, we observed that they were less likely to be asked for their opinion and actively included in the meeting.

However, even with cameras turned on we could observe challenges with the limited view of each other’s faces in digital meetings. One issue is screen sharing. This is a useful feature allowing all participants to look at the same view on the screen. However, when someone in the meeting is screen sharing, only a limited number of meeting participant faces are visible. In many of the observed meetings, screen sharing was used for an extensive part of the meeting, which limited participants’ possibility to see each other. A second issue is that the number of visible participants varies depending on the tool. For tools that displays very few faces at a time, the meeting participants who are not the most active speakers tend to disappear from the screen. We observed that this might lead to them being less likely to be invited into the discussion. Finally, a third issue is that many video conferencing platforms display the participants’ own camera image and we are not used to seeing our own faces in this way during meetings, which seemed to be distracting for some meeting participants.

## Discussion

In this article, we share our experiences from remote PPI conducted through digital meetings with contributors representing refugee parents and youth, and parents with experience of economic problems. Even though exploring remote PPI conducted through digital meetings was not an original aim of our research project, we believe that our insights during meeting observations can be of use for researchers working with involvement, including with vulnerable groups, and who are considering remote collaborations. We argue that digital PPI meetings are possible, when researchers commit to making adjustments in their preparations.

The need for remote PPI has been highlighted even before the pandemic, as it can open up for involvement for contributors who experience barriers to on-site meetings such as those who are unwell or have caring responsibilities [15]. Remote work can be done in different ways. In the observed projects in this study, video meetings have been the main collaboration tool. In relation to the Media Richness Theory, this is a relatively rich communication media, in which many of our social signals are communicated [3]. This is mainly positive, as PPI research meetings tend to be complex and large amounts of information is communicated. However, this must also be seen in the light of recent research on “Zoom fatigue” [19] which concludes that this media introduces new problems and should not be the only media used. Ideally, it should be combined with for example phone meetings to increase physical mobility [19]. In addition, different video conferencing platforms have different features, such as how many faces are visible on the screens or the option of smaller rooms within the meeting. Researchers must therefore make active choices in which media and which platform to use, depending on the meeting purpose and group involved.

One important aspect to consider is how long the team has worked together and how well the meeting participants know each other. For a new team, the communicated information should be richer, as participants have an increased need to understand the social aspects as well as the actual meeting topic. Therefore, the choice of media must reflect this and a richer media with a good ability to convey social presence [3] is to prefer. For a team with an established working relationship, the richness of the media is less important and other considerations can guide the choice.

Our insights portrayed a few potential problems with digital meetings. These included increased linguistic barriers and difficulties to claim space in a digital room, which both risk leading to contributors becoming “invisible” to others in the meeting. We also observed that digital meetings seem to offer less spontaneous positive interactions, which we believe is important for getting to know each other and building a good team [5, 18]. However, we could also see that these problems were mitigated with adaptations originating both from researchers and public contributors. One project team opted for smaller meetings with the contributors and just a few researchers, who were then responsible for representing the contributors when discussing with the larger research team. This places a large responsibility on the few researchers who met the contributors, to represent their views in a correct way. However, the smaller meeting was appreciated by the contributors, who felt that this allowed them to share their views in a comfortable way and led to a deepened discussion and experience sharing. This solution seemed to have alleviated both potential linguistic barriers and issues with speaking up in a larger meeting. Additionally, using the same interpreter in all meetings seemed to be beneficial, most likely because the interpreter also contributes to the group dynamics and sense of working as a team. This might be of increased importance when working with vulnerable groups such as the refugee population, who are less likely to become involved in research [20, 21].

In digital PPI meetings the meeting chair has an important role, possibly more important remotely than in in-person meetings. Our insights include that in digital meetings, the chair tends to, out of necessity, take a bigger and more directive role. This might be related to participants in video meetings feeling less comfortable with the format, as they are new to it, and therefore hesitate to speak up. It could indicate a need for more guidance on the expectation on and the structure of the meeting, both before and during the meeting. The meeting chair has an important role in setting ground rules that allow for all meeting participants to be included, both those that are comfortable with and those who are new with the media. One example of this is that it might be easier to use the “raise hand” function than taking the word spontaneously. The meeting chair can also advise meeting participants on how to use the screen effectively in digital meetings, for a richer interaction; keep their cameras on, minimise screen sharing, and turn off the self-view to avoid contributing to “Zoom fatigue” [19]. Additionally, making room for social interactions in a digital meeting is something that the meeting chair can contribute to, by alternating breaks away from the screen with social breaks on camera.

Recently, we have experienced that researchers who planned involvement in their projects have cancelled or postponed PPI activities, especially when involving groups with perceived barriers to involvement, such as not speaking the majority language. In one of the observed projects with contributors in the same circumstances, which included an illiterate contributor, successful involvement was possible, but detailed preparations were essential. One example of these preparations was the information material, translated to the contributors’ preferred languages, was distributed by email and by ordinary mail several weeks before the first digital meeting. The information included detailed instructions on how to download the video conferencing tool, along with how to contact the researcher for technical support if required. For some contributors, reminders were sent with an offer of guidance over phone, in their preferred language. As the research team had established email contact with all contributors—in one case with the help of a family member of a contributor—an email with guidance to join the meeting was sent hours before the meeting started. In addition to this, the interpreter was available before the meeting started for assistance with practical issues. These preparations were successful and all contributors joined the meeting. Comments from contributors included that it was challenging to access the meeting, but they were proud that they had solved it. How familiar contributors are with digital meetings is likely to differ both in and between groups. This is not something we have explored in this study; however, it appears to be important that researchers are prepared to guide contributors to the first meeting.

A recommendation for further research is to explore the experiences of both researchers and contributors involved in remote PPI activities. Additionally, involvement of contributors early in the process is preferable [7, 22] and this thinking should also be applied to digital meetings. The PPI contributors and other meeting participants should be invited to co-create the format and structure of the meeting. Starting the meeting with a short discussion on how to do it can function as a way to both raise concerns that could have been barriers to meeting success if left unattended, and to generate new ideas on how to interact remotely through digital meetings.

## Key points on conducting remote patient and public involvement

- Involving contributors from vulnerable groups should not be postponed due to the COVID-19 pandemic. With planning and adjustments, meaningful and inclusive remote involvement is feasible.
- Take an active decision on which digital media tool to use, depending on the needs, preferences and previous knowledge of the meeting participants, as well as on the meeting topic and the tools’ technical features.
- Prepare to guide your contributors to the chosen media in a way that is suitable for them. Plan for this ahead of time and be available for technical support.
- The meeting chair role is of increased importance in digital meetings, in order to facilitate an allowing atmosphere and social interactions, as well as an active inclusion of all meeting participants. The meeting chair needs to be aware of this and prepared for this responsibility.
- Seeing each other’s faces is important in digital meetings. The meeting chair should facilitate this through encouraging meeting participants to keep their cameras on and minimise screen sharing.
- Co-create the format and structure of meetings together with your meeting participants. The meeting participants are likely to have suggestions on how to make meetings more inclusive and meaningful. Be creative and open to new suggestions for collaborative work.

## Conclusions

Working remotely through digital PPI meetings are different to in-person meetings and involves a set of challenges. However, digital meetings with public contributors are possible, as long as researchers make a commitment and work together with contributors to solve practical issues. In this article, we share our insights from observations with the intention for them to be useful for other research teams. Our digital meeting observations are ongoing with the aim to explore how to conduct PPI in a meaningful way, which may include further insights on conducting PPI digitally; yet, given the shift to remote working that has been seen over the last few years and accelerated by the pandemic restrictions, further research directly addressing how to conduct PPI remotely is encouraged.

## Data Availability

Not applicable.

## Abbreviations

MRT: Media richness theory
PPI: Patient and public involvement
